# A Historical Cohort in Kidney Transplantation: 55-Year Follow-Up of 72 HLA-Identical, Donor-Recipient Pairs

**DOI:** 10.3390/jcm10235505

**Published:** 2021-11-24

**Authors:** Brian I. Shaw, Vincenzo Villani, Samuel J. Kesseli, Chloe Nobuhara, Mariya L. Samoylova, Dimitrios Moris, Bradley H. Collins, Lisa M. McElroy, Melissa Poh, Stuart J. Knechtle, Andrew S. Barbas, Hilliard F. Seigler

**Affiliations:** 1 Department of Surgery, Duke University, Durham, NC 27710, USA; vincenzo.villani@duke.edu (V.V.); samuel.kesseli@duke.edu (S.J.K.); mariya.samoylova@duke.edu (M.L.S.); dimitrios.moris@duke.edu (D.M.); bradley.collins@duke.edu (B.H.C.); lisa.mcelory@duke.edu (L.M.M.); stuart.knechtle@duke.edu (S.J.K.); andrew.barbas@duke.edu (A.S.B.); hilliard.siegler@duke.edu (H.F.S.); 2 School of Medicine, Duke University, Durham, NC 27710, USA; chloe.nobuhara@duke.edu; 3 Department of Plastic Surgery, University of Southern California, Los Angeles, CA 90033, USA; melissa.poh@gmail.com

**Keywords:** kidney transplant, long-term outcomes, HLA matching, precision transplant

## Abstract

The impact of HLA matching on graft survival has been well characterized in renal transplantation, with a higher degree of matching associated with superior graft survival. Additionally, living donor grafts are known to confer superior survival compared to those from deceased donors. The purpose of this study is to report our multi-decade institutional experience and outcomes for patients who received HLA-identical living donor grafts, which represent the most favorable scenario in kidney transplantation. We conducted a retrospective analysis of these graft recipients performed at a Duke University Medical Center between the years of 1965 and 2002. The recipients demonstrated excellent graft and patient survival outcomes, superior to a contemporary cohort, with median patient and graft survival of 24.2 and 30.9 years, respectively, among Duke recipients vs. 16.1 and 16.0 years in a cohort derived from national data. This study offers a broad perspective on the importance of HLA matching and graft type, and demonstrates a historical best-case-scenario in renal transplantation.

## 1. Introduction

The importance of histocompatibility has been recognized since the inception of transplantation surgery as a field, even prior to the elucidation of human leukocyte antigen (HLA) in 1958 by Jean Dausset [1,2]. In 1954, a living donor kidney transplant (LDKT) performed by Merrill and Murray et al. between a pair of identical twins became widely publicized as the first successful human kidney transplant [3]. The 24-year-old recipient in this case ultimately survived an additional 8 years, far longer than previous attempts in non-twin pairs from the same era, who survived on the order of days to months [4]. Later, in 1960, Merrill reported a successful kidney transplantation in non-identical twins with matched blood group antigens [5].

The Duke kidney transplant program began in 1965 with an intentional focus on selecting HLA-identical donor-recipient pairs using a combination of skin grafting, mixed lymphocyte reactions, and microcytotoxicity assays [6]. The first 16 such patients were treated with 50 mg azathioprine daily as sole immunosuppressant with variable outcomes, and 9 of these patients were treated with steroids for rejection episodes. Further studies demonstrated good outcomes, but with an increase in rejection episodes among patients who were haploidentical as compared to genetically identical [7], with excellent medium-term outcomes up to ten years [8]. The initial reported experience led to many subsequent improvements, including intensified immunosuppressive treatment and expansion into HLA-mismatched living donor and deceased donor transplants.

Since then, the field of kidney transplantation has evolved considerably. Changes in donor selection, organ preservation, and, most importantly, immunosuppression have transformed kidney transplantation from an experimental therapy to a routinely performed procedure for patients with end-stage renal disease. Although the first successful clinical kidney transplant was performed in living identical siblings [3], in the US deceased donors now represent the primary source of organs, making up about two-thirds of the total kidney transplant volume [9]. Several authors have investigated the outcomes of LDKT vs. deceased donor kidney transplantation (DDKT), with overwhelming evidence that living donation is associated with superior patient and graft survival [9,10,11].

HLA matching was once considered an essential factor in donor selection based on the association of mismatch with deceased donor allograft survival [12,13,14]. Currently, HLA matching in the United Network for Organ Sharing (UNOS) is performed by typing recipient and donor HLA at the HLA-A, -B, and -DR loci. Therefore, the best match a patient may have is 0/6 (2 alleles per loci) [15]. However, advances in immunosuppression have diminished the negative consequences of HLA mismatch [16], such that it is regarded as less relevant in contemporary matching schemes [17]. In LDKT, some studies have reported no influence of HLA matching on graft survival [18], while others have demonstrated an increasing hazard of graft loss with a greater number of mismatches [19]. Recent work has shown that HLA matching may indeed be important even in contemporary cohorts when examining physical similarities between HLA, rather than simply allele mismatch, termed “eplet” matching [20].

Having performed kidney transplantation since 1965, the Duke University Medical Center is home to a unique cohort of HLA-matched LDKT recipients with ultra-long term follow up. Given the historical and ongoing relevance of HLA matching in transplantation, the purpose of this study is to characterize our multi-decade institutional experience and outcomes for patients who received what is considered the most optimal graft: HLA-identical kidneys from a living donor.

## 2. Materials and Methods

### 2.1. Cohort Construction

This study is a retrospective analysis of HLA-identical living donor kidney transplants performed at a single institution between the years of 1965 and 2002. Of the 2262 renal transplants performed at this institution during this time period, 118 allografts were utilized from HLA-identical donors. Of these 118 patients transplanted, 23 patients were excluded due to lack of patient survival data, 18 were excluded for lack of graft survival data, 3 were excluded due to lack of immunosuppression data, and 2 were excluded due to having a known deceased donor after further review. We were therefore able to obtain complete records and include 72 of those individuals. Of special note, HLA typing in this cohort was robust, with 35/72 (49%) having sufficient familial genotypic information to ensure that donor and recipient were HLA genetically identical. For donors about whom this degree of genetic information was not available, they were considered serologically identical using the serological techniques available at the time.

Additionally, we constructed two cohorts using the UNOS Standard Transplant Analysis and Research (STAR) file to examine approximate patient and graft survival for both a historical cohort (relative to our experience) and a modern cohort. For the historical cohort, we included all patients transplanted prior to 2003 (185,271) with an age greater than 14 (the minimum age in our cohort, 179,086), who had no prior transplants (131,853), who received only a kidney (118,305) from a living donor (21,620). For the modern cohort, we examined all patients transplanted after 2003, using the same criteria that was used for the historical cohort, which yielded a total of 73,623 transplant recipients. As a sensitivity analysis, we determined the death-censored graft survival in a subset of our UNOS cohort, including only patients who had 0/6 HLA mismatches at HLA-A, -B, and -DR.

### 2.2. Primary Outcome

We examined patient- and death-censored graft survival in this cohort from the time of transplant until 31 March 2019.

### 2.3. Analysis

Patient characteristics were summarized using appropriate measures of central tendency. Patient- and death-censored graft survival were plotted using Kaplan–Meier curves. Median survivals were determined with 95% confidence intervals.

## 3. Results

### 3.1. Cohort Description 

Seventy-two (72) patients from our cohort met the inclusion criteria. Of this total, 31 (44%) were female. The cohort was young (median age 40, IQR 30–52), mostly white (74%), and the most common cause of renal disease was chronic glomerulonephritis. Many patients were on hemodialysis (42%) at the time of transplant or had been on dialysis for a short duration (median of 6 months, IQR 2–14 months). Most patients were initially managed on azathioprine and nearly all received steroids (Table 1). Of note, three patients were able to discontinue all immunosuppression; one of these had received a transplant from an identical twin sibling.

**Figure 1:**
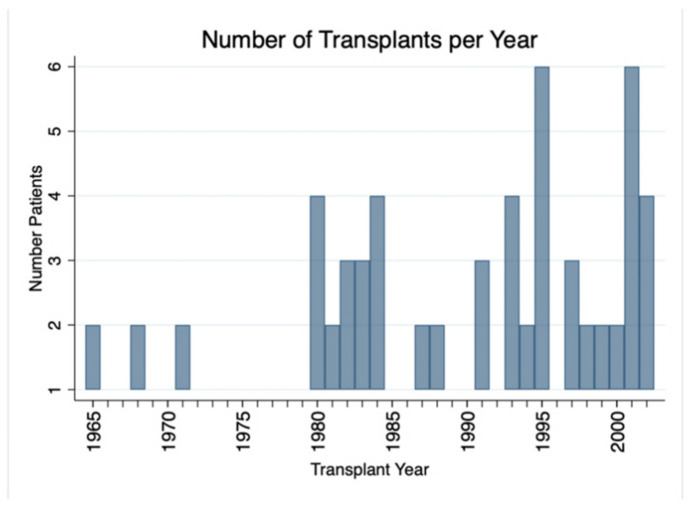
Distribution of transplants by date. Genetically identical transplants were performed between 1965 and 2002.

Though detailed donor information was not available, the majority (69/72) were living related donors with only 3 living unrelated donors. The cohort was transplanted between 1965 and 2002; the distribution of transplants over time is shown in Figure 1.

### 3.2. Patient Outcomes 

**Figure 2:**
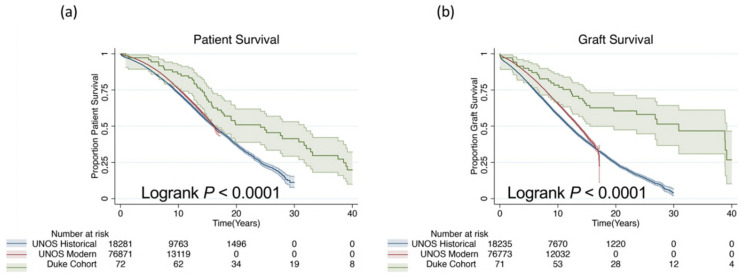
Patient and graft survival. Patient- (**a**) and death-censored graft (**b**) survival are depicted using the Kaplan–Meier method. Shading is 95% CI.

Overall, 10-year patient and graft survival were 86% and 77%, respectively (Figure 2). The median patient survival was 24.2 years (95% CI 16.7–31.7) and median graft survival was 30.9 years (95% CI 15.5–39.1). We examined UNOS data to compare our cohort to both a historical cohort (transplants prior to 2003) and a modern cohort (transplants in 2003 and thereafter) of living donor kidney transplant recipients (Table S1). Overall, our HLA-identical cohort demonstrated superior survival compared to both a historical cohort and a modern cohort (log-rank *p* < 0.001 for both). As a sensitivity analysis, we compared death-censored graft survival among patients with 0/6 HLA mismatches in the UNOS cohorts relative to the Duke cohort. The median death-censored graft survival in the historical UNOS cohort was 16.0 years (95% CI 14.8–16.5) and 16.9 years (95% 16.1–∞) in the modern UNOS cohort compared to over 30 years in the Duke cohort.

Though data were limited and there were no consistent biopsy protocols over the full time period, 23/72 (32%) of our cohort experienced acute rejection within the first year of transplant. This number decreases slightly to 17/72 (24%), if including only biopsy-proven rejection. Overall, 50% of patients died with a functioning graft.

## 4. Discussion

Historically, living related donors comprised the initial supply of kidney allografts; however, over time, due to ethical considerations and scale of demand, unrelated deceased donors have become the predominant source [21]. As our knowledge of HLA has increased and immunosuppression has improved, HLA matching has become less prioritized in transplantation [17]. However, in the present study, we present a historical cohort that demonstrated excellent graft and patient survival in the context of very specific and precise HLA matching, which is distinctly different than the HLA matching used by UNOS today.

It is well-established that grafts utilized from living-related donors demonstrate a longer median survival than those recovered from deceased donors (16 vs. 9 years among the youngest donors and recipients, age 18–39) [22]. Furthermore, a recent study of 143 adult kidney transplants performed between HLA-identical twins showed remarkably high recipient and graft survival rates at 1, 3, 5, and 10 years post-transplant (98.6%, 97.8%, 97.8%, and 95.4% versus 97.2%, 93.5%, 91.9%, and 83.9%, respectively) [23]. In the present study, the median graft survival was 30 years with 61% of grafts surviving at least 20 years, significantly longer than that of the overall living donor pool. This is likely attributable to the high degree of HLA-antigen matching. Indeed, the majority of donors for whom we have data in this cohort were family members of the transplant recipients. Given our increasing understanding of the complexity of HLA, with a large number of new genetic variants recently discovered due to advances in DNA sequencing, it is unsurprising that matching between family members might be superior to matching based on serological typing [24]. HLA matching in UNOS is still pursued through serological-equivalent typing even where sequence-based typing is available. This suggests that the typing used by UNOS is less accurate than it could be. Additionally, increasing evidence suggests that the physical dissimilarity between HLA and not just disparate typing (either serologic or molecular) contribute the most to the immunological risk of HLA mismatch [20]. Though the present study does not specifically answer the question of whether more weight should be placed on HLA matching in current organ allocation, it does suggest that very favorable long-term outcomes may be achieved when high degrees of HLA matching are pursued, even in a historical setting.

One interesting aspect of this analysis is the high incidence of early acute rejection events in the Duke cohort. At least 24% (17/72) of patients experienced biopsy-proven acute rejection within the first year. In spite of this, patients had excellent survival and graft outcomes. Recently, a multi-center retrospective analysis showed an incidence of 12.9% of biopsy-proven acute rejection in living donor HLA-identical kidney transplant recipients, with an average occurrence within 24 months after transplantation. In their study, factors associated with rejection were recipient age (OR, 0.91 (0.84–0.96); *p* = 0.003), donor BMI (odds ratio (OR), 1.22 *1.04–1.46); *p* = 0.01), and minimization of immunosuppression (OR, 26.2 (5.48–166.6); *p* < 0.001). Of interest, patient and graft survival rates were not statistically different according to rejection at 1, 5, and 10 years post-transplant [25]. Multiple studies across diverse cohorts have shown an association between acute rejection and worse death-censored graft survival, even when examining subclinical rejection [26,27,28]. However, certain populations do not show this association, such as in patients managed on immunosuppression using co-stimulation blockade who have superior graft survival compared to patients managed on calcineurin inhibitors in spite of multiple early acute rejection episodes [29,30]. These disparate results suggest that specific contexts may mediate the relationship between acute rejection and long-term outcomes. It is possible that the lack of association seen in the present cohort is due to either the non-CNI based immunosuppression utilized in many patients, the greater genetic similarity between donor and recipient, or other systems factors that allowed for prompt and adequate treatment of rejection episodes.

One aspect of transplantation this study highlights is the potentially salutatory effects of kidney exchanges. Kidney exchange mechanisms allow for the broader sharing of grafts from living donors in order to improve overall “compatibility”—including HLA matching—between donor and recipient [31]. Indeed, in silico simulations have shown that exchange programs have the potential to decrease HLA mismatch between donor and recipient [32].

There are several limitations in this study that deserve mentioning. First, we recognize our cohort is a small, select group of ideal recipients from a center with a large transplant experience. However, this does not negate the finding that these outcomes exceeded those in both historic and contemporary cohorts. We also did not control for demographic differences between the UNOS-derived cohorts and those from our institution, which limits comparisons. Additionally, we were only able to obtain complete follow-up information on a fraction (~60%) of the total cohort. However, the included and excluded patients were relatively similar, differing only in age at transplant (those included were older), azathioprine usage (less in the included), and mycophenolate usage (more in the included group). The HLA typing in this cohort is distinct from that used by UNOS during the majority for the study and therefore limits formal comparisons. However, it also presents and opportunity to examine exceedingly well-matched individuals outside of the three HLA loci generally reported to UNOS. Additionally, any analysis of acute rejection events in this cohort is limited both by the vast changes in technology and terminology that occurred over the interval in which this cohort was observed. The first Banff Conference on transplant pathology—the modern institution for standardizing pathologic analysis of transplant specimens—was not held until 1991. However, given the fact that there was likely underdiagnosis of rejection events, the lack of association between rejection and poor outcomes, which comports with other contemporary data, is of interest.

## 5. Conclusions

In conclusion, this report represents a unique analysis of patients who underwent HLA-identical renal transplantation at a single center with ultra-long follow up. Although the relationship between graft survival and rejection rates with an increasing degree of HLA match is well established, this study offers a broad historical perspective on the importance of HLA matching. Additionally, the observation of very favorable graft outcomes in spite of relatively high levels of acute rejection may indicate a moderation of the deleterious effects of acute rejection in genetically similar individuals.

## Figures and Tables

**Table 1:** Duke Cohort Demographics.

	Included
	*N* = 72
**Gender (Female)**-*n* (%)	31 (43%)
**Race**	
Black	17 (24%)
Other	2 (3%)
White	53 (74%)
**Age at Transplant**-Med (IQR)	40 (30–52)
**Diagnosis**-*n* (%)	
Chronic Glomerulonephritis	27 (39%)
Hypertension	8 (11%)
Diabetes	8 (11%)
Obstructive Uropathy	4 (6%)
Lupus	5 (7%)
Polycystic Kidney Disease	4 (6%)
Post-streptococcal Glomerulonephritis	5 (7%)
Other	9 (13%)
**Dialysis Type**-*n* (%)	
Peritoneal Dialysis	11 (15%)
Hemodialysis	30 (42%)
Both	8 (11%)
No Dialysis	8 (11%)
Unknown	15 (21%)
**Duration of Dialysis (Months)**-Med (IQR)	6 (2–14)
**Immunosuppression (Ever Used)**-*n* (%)	
Azathioprine	47 (65%)
Prednisone	69 (96%)
Cyclophosphamide	4 (6%)
Mycophenolate Mofetil	24 (33%)
Calcineurin Inhibitor	29 (40%)

## Data Availability

Data available upon request from the authors.

## Supplementary Materials

The following are available online at https://www.mdpi.com/article/10.3390/jcm10235505/s1, Table S1: UNOS Cohort Descriptions.
