# Supplementary material for: A Historical Cohort in Kidney Transplantation: 55-Year Follow-Up of 72 HLA-Identical, Donor-Recipient Pairs
Source: J Clin Med. 2021 Nov 24;10(23):5505. doi: 10.3390/jcm10235505 (PMC8658388; doi:10.3390/jcm10235505)
Supplement: Supplementary file 1 [file jcm-10-05505-s001.zip › jcm-1447639-supplementary.pdf]

Supplemental Table S1: UNOS Cohort Descriptions

	Historic UNOS Cohort N=18,287	Modern UNOS Cohort N=76,956	p-value
**Gender (Female)-**n(%)	7,539 (41%)	29,276 (38%)	<0.001
**Age**-Med(IQR)	43.0 (32.0-53.0)	50.0 (38.0-59.0)	<0.001
**Race/Ethnicity**-n(%)			<0.001
White	11,942 (65%)	49,279 (64%)	
Black	3,072 (17%)	11,087 (14%)	
Hispanic	2,208 (12%)	11,509 (15%)	
Asian	712 (4%)	3,865 (5%)	
Native American	154 (1%)	477 (1%)	
Native Hawaiian	87 (0%)	243 (0%)	
Multiracial	110 (1%)	496 (1%)	
Unknown	2 (0%)	0 (0%)	
**Dialysis Time(mos)**-Med(IQR)	14 (8-25)	16 (8-29)	<0.001
**On Dialysis?**-n(%)	14,490 (81.9%)*	51,018 (66.3%)*	<0.001
**BMI**-Med(IQR)	24.9 (22.0-28.4)^	27.3 (23.7-31.4)*	<0.001
**Diagnosis**-n(%)			<0.001
Cystic/Congenital	1,553 (11%)&	11,478 (15%)*	
Diabetic Nephropathy	3,374 (23%)&	17,495 (23%)*	
Glomerulonephritis	4,609 (31%)&	21,851 (29%)*	
Hypertension	2,279 (15%)&	13,146 (17%)*	
Obstructive Uropathy	581 (4%)&	2,121 (3%)*	
Other	2,348 (16%)&	10,540 (14%)*	

* <5% missing; ^ 5-10% missing; &10-20% missing
